# Context of walking and loneliness among community-dwelling older adults: a cross-sectional study

**DOI:** 10.1186/s12877-023-04043-5

**Published:** 2023-05-25

**Authors:** Sachiko Mizuta, Kazuaki Uchida, Ryuichi Sawa, Junya Nakamura, Haruhi Encho, Toshihiro Akisue, Rei Ono

**Affiliations:** 1 Department of Rehabilitation Science, Kobe University Graduate School of Health Sciences, Hyogo, Japan; 2 Department of Prevention and Care Science, Research Institute, National Center for Geriatrics and Gerontology, Aichi, Japan; 3 Department of Physical Therapy, Faculty of Health Science, Juntendo University, Tokyo, Japan; 4 Department of Dentistry and Oral Surgery, National Center for Geriatrics and Gerontology, Aichi, Japan; 5 National Institute of Health and Nutrition, Health and Nutrition, National Institutes of Biomedical Innovation, KENTO Innovation Park NK Bldg., 3-17, Senriokashinmachi, Settu, Osaka 566-0002 Japan

**Keywords:** Loneliness, Walking, Mental health, Physical activity, Older adults

## Abstract

**Background:**

Older adults are more likely to experience loneliness than younger people. Moreover, greater loneliness in older adults is associated with poor mental health and increased risk of cardiovascular disease and mortality. Physical activity is an effective intervention for reducing loneliness among older adults. Among physical activities, walking is suitable for older adults, because it is easy and safe to incorporate into daily life. We hypothesized that the association between walking and loneliness depends on the presence of others and the number of people present. The aim of this study is to investigate the association between the context of walking (the number of walkers) and loneliness among community-dwelling older adults.

**Methods:**

This cross-sectional study included 173 community-dwelling older adults aged 65 or older. Context of walking was classified as non-walking, walking alone (days of walking alone > days of walking with someone), and walking with someone (days of walking alone ≤ days of walking with someone). Loneliness was measured using the Japanese version of the University of California Los Angeles Loneliness Scale. A linear regression model was used to investigate the relationship between context of walking and loneliness, adjusted for age, sex, living situation, social participation, and physical activity excluding walking.

**Results:**

Data from 171 community-dwelling older adults (mean age = 78.0 years, 59.6% women) were analyzed. After adjustment, walking with someone was associated with lower loneliness than non-walking (adjusted β: -0.51, 95% confidence interval: -1.00, -0.01).

**Conclusions:**

The study’s findings suggest that walking with a companion may effectively prevent or reduce loneliness among older adults.

## Introduction

Loneliness refers to the subjective experience derived from the discrepancy between desired and actual social relationships [1], and differs from social isolation, the objective absence or lack of contacts and interactions between family, friends, and community [2]. Older adults are more likely to experience loneliness because of the loss of family and friends and a lack of relationships [3–7]. Greater loneliness in older adults is associated with poor mental health and an increased risk of cardiovascular disease and mortality [8–11]. Therefore, there is an increasing need for solutions to prevent or reduce loneliness among older adults.

Previous systematic reviews have reported that group-based psychological, technology-based, and exercise-based interventions were effective in reducing loneliness [12, 13]. Among the exercise-based interventions, walking is suitable for older adults because it is easy to incorporate into daily activities, regardless of age and sex and with a low risk of injury, and it does not require special equipment [14, 15]. A longitudinal study that investigated the association between walking and loneliness reported that older adults who spent more time walking per week showed lower levels of loneliness over three years [16]. Recent studies have focused on context of walking, such as the number of people walking together. Previous studies have reported that walking with someone has more positive effects on increasing physical activity (PA), motivation, and self-efficacy [17–19]. To date, it has been suggested that others’ presence is important to reduce loneliness [20], and walking with someone can affect psychosocial aspects [21]. Therefore, we hypothesized that walking with someone would be associated with a decrease in loneliness. Investigating the relationship between context of walking and loneliness may provide effective solutions to prevent or reduce loneliness among older adults. However, no study has yet investigated this association.

This study aimed to investigate the association between context of walking (non-walking, walking alone, and walking with someone) and loneliness among community-dwelling older adults.

## Methods

### Study design and participants

This cross-sectional study included community-dwelling older adults who were invited to participate in health check-ups. Participants were recruited with the cooperation of community associations and senior citizens’ associations in Suma-ku, Kobe, Japan. Specifically, flyers for the health check-up were distributed to the members of each association, or posted on the bulletin board of the community welfare centre. The health check-up was open to all older adults aged 65 years or older who were able to visit the health check-up site. A total of 173 Japanese community-dwelling older adults aged 65 years or older participated in the health check-up. Data were collected by trained physical therapists or physical therapy students at the health check-ups, conducted between September and October, 2021. During the health check-up, participants’ physical and cognitive functions were measured and questionnaires were completed. To ensure consistency of measurements, a personnel member in charge was assigned to each measurement. Exclusion criteria were missing data (*n* = 2). This study was approved by the ethics committee of Kobe University (authorization number 625-4), and all participants provided written informed consent.

### Loneliness

Loneliness was assessed using the Japanese version of the University of California Los Angeles (UCLA) Loneliness Scale [22, 23]. This scale comprises 20 questions, with a four-point scale ranging from “never” (score of 1) to “always” (score of 4). The total scores ranged from 20 to 80, with higher scores indicating greater loneliness. The UCLA Loneliness Scale is a self-completion questionnaire that has been proven to be reliable (Cronbach’s alpha = 0.92) among Japanese older adults [22].

### Context of walking

First, participants were asked, ‘Excluding walking for work or transportation, generally, how many days in a week do you walk for at least 10 minutes at a time in your leisure time?’ This is included in the Japanese long version of the International Physical Activity Questionnaire (IPAQ) [24]. Participants who walked once a week or more were then asked the following question: ‘How many days per week did you go for a walk with a partner, friend, or as part of a group?’ [18]. The context of walking was classified into one of three groups: non-walking (W0), walking alone (days of walking alone > days of walking with someone, W1), and walking with someone (days of walking alone ≤ days of walking with someone, W2) [25].

### Other variables

Demographic data, including age, sex, years of education, body mass index (BMI; calculated as kg/m^2^), and living situation (living with someone or alone) were recorded. Social participation was defined as participation in at least one of the six types of group activities or associations at least once a week: senior citizens’ club, sports-related group, hobby-related group, volunteer or non-profit organization group, residents’ association, and religious groups [26]. Walking time per day was assessed using the Japanese long version of the IPAQ [24]. “PA excluding walking” was calculated as the total sum of the moderate and vigorous-intensity PA per week. Moderate and vigorous-intensity PA were calculated by multiplying the number of days per week, the total number of minutes per day, and metabolic equivalents (METs) corresponding to the intensity of each PA (moderate-intensity PA, four METs; vigorous-intensity PA, eight METs) [27].

### Statistical analysis

To compare the differences between context of walking, we performed ANOVA, Kruskal–Wallis test, chi-squared (X^2^) test, and a Mann-Whitney U test. We used a post hoc Steel-Dwass test for variables that showed significant differences.

A linear regression model was used to investigate the association between context of walking and loneliness. The dependent variable was the UCLA loneliness score, and the independent variable was context of walking. Context of walking was defined as a categorical variable. The following variables were treated as confounding factors in the multivariate model: age, sex, living situation, social participation, and PA excluding walking. All confounding factors were selected based on previous studies [3, 28, 29]. Beta values (β), 95% confidence intervals (95% CI), and *p*-values were estimated for both models. Statistical significance was set at *p* < 0.05, and all statistical analyses were conducted using Jamovi software (version 2.2.2; The Jamovi Project, Sidney, Australia).

## Results

### Characteristics of participants

Table 1 presents the characteristics of participants in this study. A total of 171 older adults aged 65 years or older were included. The mean age ± standard deviation was 78.0 ± 5.6 years, and the percentage of women was 59.6% (*n* = 102). The percentage of older adults regarding context of walking (W0, W1, and W2) was 24.6% (*n* = 42), 60.2% (*n* = 103), and 15.2% (*n* = 26), respectively. There were no significant differences in the days of walking between W1 and W2 (mean days ± SD: W1, 4.6 ± 1.9; W2, 4.5 ± 2.1; *p* = 0.86) or walking time (mean time ± SD: W1, 65.2 ± 48.9; W2, 83.5 ± 58.6; *p* = 0.06). Participants in W2 have significantly lower loneliness, compared with those in W0 (W0, 38.2 ± 9.2; W1, 36.2 ± 9.9; W2, 31.9 ± 7.0; post hoc Steel-Dwass test, *p* = 0.02).

**Table 1:** Characteristics of participants

Variable	Total (*N* = 171)	Context of walking ^a^			*P-*value ^d^
		W0 (*n* = 42)	W1 (*n* = 103)	W2 (*n* = 26)	
Age, years	78.0 ± 5.6	77.6 ± 7.1	78.1 ± 5.3	78.4 ± 4.5	0.81
Women, *n* (%)	102 (59.6)	28 (66.7)	58 (56.3)	16 (61.5)	0.50
Years of education, years	12.7 ± 2.5	12.5 ± 2.8	12.9 ± 2.5	12.3 ± 2.3	0.60
BMI, kg/m²	23.1 ± 2.8	23.1 ± 3.2	23.1 ± 2.6	23.4 ± 3.0	0.86
Living with someone, *n* (%)	121 (70.8)	30 (71.4)	68 (71.4)	23 (88.5)	0.08
Social participation ^b^, *n* (%)	107 (62.6)	23 (54.8)	62 (60.2)	22 (84.6)	0.04 ^e^
PA excluding walking ^c^ (METs minute/week)	1372.4 ± 2334.4	870.5 ± 2020.8	1405.0 ± 2356.2	2053.8 ± 2607.4	0.04
Days of walking, day/week	-	-	4.6 ± 1.9	4.5 ± 2.1	0.86
Walking time, minute/day	-	-	65.2 ± 48.9	83.5 ± 58.6	0.06
UCLA loneliness scale, scores	36.0 ± 9.5	38.2 ± 9.2	36.2 ± 9.9	31.9 ± 7.0	0.02 ^e^

Data described as *n* (%) or mean ± standard deviation

Abbreviations: BMI = body mass index; PA = physical activity; UCLA = University of California, Los Angeles

^a^ Context of walking was classified into three groups: non-walking (W0), walking alone (W1), and walking with someone (W2)

^b^ Social participation was defined as the number of people who participated at least once a week in any of the social groups or associations

^c^ PA excluding walking was defined as the sum of moderate and vigorous PA

^d^ W0, W1, and W2 were compared using ANOVA, Kruskal–Wallis test, and chi-squared test. W1 and W2 were compared using the Mann-Whitney U test

^e^ Significant difference between W0 and W2 using post hoc Steel-Dwass test

**Fig. 1:**
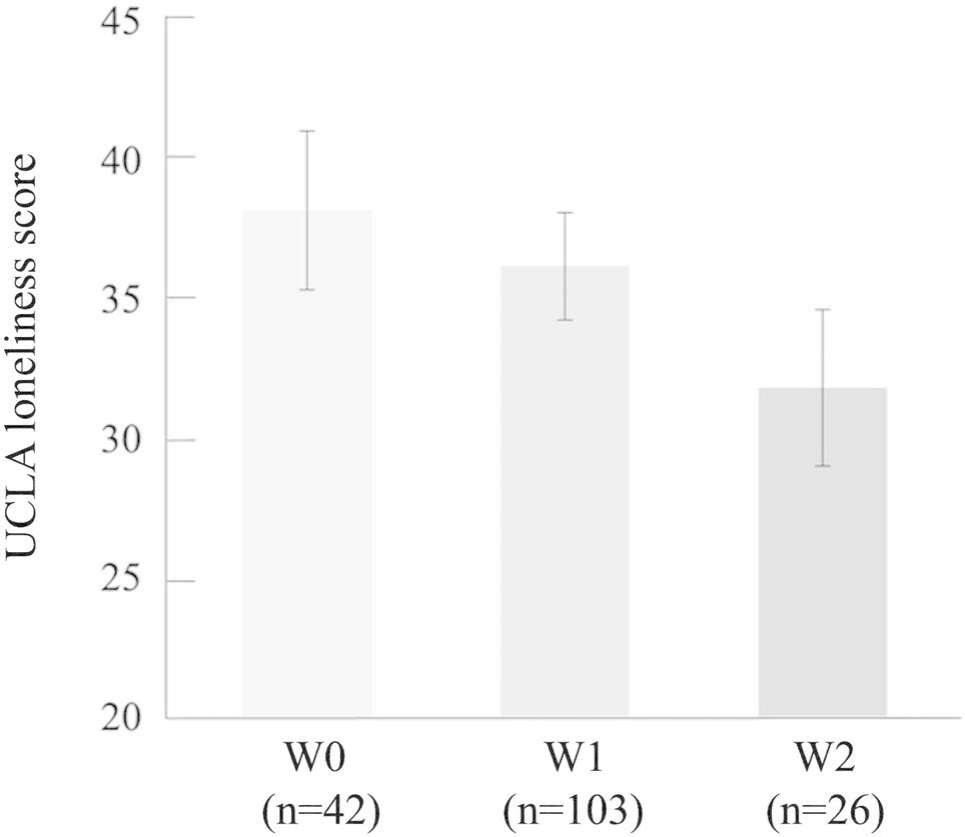
Distribution of UCLA loneliness score divided by context of walking. Data are presented as mean with 95% confidence interval. Context of walking was classified into three groups: non-walking (W0), walking alone (W1), and walking with someone (W2)

Figure 1 shows the UCLA loneliness scores among the three groups, categorized by context of walking, indicating that the UCLA loneliness score decreases in the order of W0, W1, and W2.

### Associations between context of walking and loneliness

Table 2 shows the results of the linear regression analysis of the associations between context of walking and UCLA loneliness score. In the univariate model, W2 was significantly associated with lower loneliness than W0 (β: -0.67, 95% CI: -1.16, -0.19, *p* < 0.01), but not with W1 (β: -0.21, 95% CI: -0.57, 0.14, *p* = 0.24). Even after adjustment, W2 was significantly associated with lower loneliness than W0 (β: -0.51, 95% CI: -1.00, -0.01, *p* = 0.04).

**Table 2:** Associations between Context of walking and UCLA loneliness score: Linear regression analysis

Description	Univariate model			Multivariate model ^b^		
	β	95% CI	*P-*value	β	95% CI	*P-*value
Context of walking ^a^						
W0	ref.			ref.		
W1	-0.21	-0.57, 0.14	0.24	-0.22	-0.57, 0.14	0.23
W2	-0.67	-1.16, -0.19	< 0.01	-0.51	-1.00, -0.01	0.04
Age				0.01	-0.14, 0.17	0.86
Sex				-0.22	-0.57, 0.13	0.22
Living situation				-0.37	-0.75, 0.001	0.05
Social participation				-0.14	-0.46, 0.18	0.38
PA excluding walking				-0.14	-0.30, 0.02	0.08

Abbreviations: PA = physical activity; β = standardized partial regression coefficient; 95% CI = 95% confidence interval

^a^ Context of walking was classified into three groups: non-walking (W0), walking alone (W1), and walking with someone (W2)

^b^ Adjusted for age, sex, living situation (living with someone or living alone), social participation, and PA excluding walking

## Discussion

This cross-sectional study investigated the association between context of walking and loneliness among community-dwelling older adults. The results showed that loneliness was significantly lower when walking with someone, whereas there was no significant difference between walking alone and non-walking.

This is the first study to investigate the association between walking and loneliness, focusing on others’ presence.

Gyasi et al. [30] reported that older adults who engaged in more days of PA in a week had lower loneliness, suggesting that the association between PA and lower loneliness may be strengthened by social connectedness. This finding suggests that the association between PA and loneliness may depend on whether physical activity was conducted with someone or alone. However, they did not obtain information on the number of people engaged in PA. Therefore, we investigated the association between context of walking and loneliness and found that walking with someone was associated with lower loneliness. This study extends the findings of previous studies and suggests the importance of PA engaging with others to reduce loneliness.

Previous meta-analyses have reported that PA increases self-efficacy in older adults [31]. Greater self-efficacy is associated with lower loneliness [32]. The positive effect of PA on self-efficacy may reduce loneliness, as older adults with greater self-efficacy are more likely to have positive thoughts and undertake activities [33]. Furthermore, it has been suggested that self-efficacy may be enhanced when engaging in PA with someone [34].

Moreover, PA with someone provides more opportunities for social interaction, compared to PA alone [35]. These PAs seem to offer diverse relationship opportunities and facilitate a sense of belonging to community and group members [13]. The tripartite model of group identification, which is one of the loneliness reduction models, suggests that having a sense of identification and social attraction to group members with shared interests and goals during PA is effective in reducing loneliness [36]. Thus, the results may be explained by the positive effects of walking with someone on self-efficacy and social interaction.

Those who engage in more frequent PA with someone have more opportunities to benefit from the positive effect of self-efficacy and social interaction, compared with those who engage in more frequent PA alone. Previous studies concerning subjective health and mental health status have also reported the importance of increasing the frequency of exercise with someone [25, 37], and this study reported similar findings.

Walking with someone is easy to incorporate into daily life, even for older adults who are unfamiliar with exercise. This finding suggests that it is one of the effective solutions to prevent or reduce loneliness among older adults.

This study had some limitations. First, because it used a cross-sectional design, a longitudinal study is required to investigate the impact of walking with someone on loneliness. Second, the impact of the coronavirus disease 2019 (COVID-19) pandemic was not considered in this study. Although there were no instructions to limit interactions or social activities with others, the COVID-19 pandemic may lead to increased loneliness due to reduced opportunities for face-to-face activities such as walking with someone. Third, this study did not ask participants whom they walked with (e.g., friends or family). Loneliness may be influenced by the closeness of the person walking, and this information needs to be obtained in future studies. Fourth, participants had lower mean UCLA loneliness scores compared to the mean UCLA loneliness score of 42.2 ± 9.9 among randomly selected older adults in a previous study [22]. This difference may be because the participants in this study were relatively healthy older adults recruited from community clubs who had more opportunities for daily contact with friends and neighbors. Therefore, caution must be exercised when generalizing these results. However, considering the impact of social interaction on daily living beyond walking, this study adjusted for social participation as a confounding factor. Fifth, PA was assessed using a self-administered questionnaire. Therefore, the assessment may not precisely reflect actual PA. Future studies should use objective indicators. Sixth, we were unable to adjust for other confounders that may influence loneliness. However, we have selected confounding factors that are particularly suggested to influence loneliness, based on previous studies [3, 28, 29].

In conclusion, this study investigated the association between context of walking and loneliness among community-dwelling older adults. The findings suggest that walking with someone is one of the effective solutions to prevent or reduce loneliness among older adults.

## Data Availability

The datasets generated and/or analyzed during the current study are not publicly available due to contain information that could compromise the privacy of research participants but are available from the corresponding author on reasonable request.

## Abbreviations

PA: physical activity
UCLA: University of California, Los Angeles
BMI: body mass index
METs: metabolic equivalents
95% CI: 95% confidence intervals
